# Extracellular CIRP augments inflammation in acute kidney injury via NKG2D-positive macrophages

**DOI:** 10.3389/fimmu.2025.1703126

**Published:** 2026-01-08

**Authors:** Fangming Zhang, Hui Jin, Chuyi Tan, Ping Wang, Max Brenner

**Affiliations:** 1 Center for Immunology and Inflammation, The Feinstein Institutes for Medical Research, Manhasset, NY, United States; 2 Departments of Surgery and Molecular Medicine, Zucker School of Medicine at Hofstra/Northwell, Manhasset, NY, United States

**Keywords:** AKI, eCIRP, macrophages, MULT-1, NKG2D, RIR, RTECs

## Abstract

**Introduction:**

The mechanism by which extracellular cold-inducible RNA-binding protein (eCIRP) aggravates renal ischemia/reperfusion (RIR) injury leading to acute kidney injury (AKI) is poorly understood. The natural killer group 2D (NKG2D) receptor and its ligand MULT-1 are key immunoregulatory mechanisms promoting responses to damaged and inflamed cells.

**Methods:**

We subjected wild-type and CIRP^−/−^ mice to RIR. We then used immunohistochemistry (IHC), flow cytometry, and Western blotting to assess NKG2D and MULT-1 in kidney tissues, macrophages, and renal tubular epithelial cells (RTECs), and ELISA to assess TNFa and IL-6.

**Results:**

The expression levels of NKG2D and its ligand MULT-1 were significantly elevated in wild-type mice subjected to RIR compared with sham. In contrast, CIRP^−/−^ mice exhibited markedly reduced expression of both NKG2D and MULT-1 after RIR compared to wild-type mice. *In vitro*, eCIRP stimulated the expression of NKG2D in peritoneal macrophages and of MULT-1 in RTECs. Treatment of eCIRP-stimulated peritoneal macrophage and RTEC co-cultures with an NKG2D-neutralizing antibody significantly and markedly downregulated supernatant levels of TNFa and IL-6.

**Discussion:**

In conclusion, eCIRP induces NKG2D^+^ macrophages and MULT-1^+^ RTECs, and their interaction further increases the inflammatory response. Targeting the NKG2D/MULT-1 may reduce RIR-induced inflammation and thus attenuate AKI.

## 1 Introduction

Acute kidney injury (AKI) is a common and potentially deadly abrupt decline in renal function characterized by reduced excretory function (increased creatinine and decreased glomerular filtration rate), disordered electrolytes (hyperkalemia), and disrupted fluid homeostasis (oliguria) (1). A major cause of AKI is renal ischemia-reperfusion (RIR) resultant of renal hypoperfusion in the contexts of shock (e.g., hypovolemia, peripheral vasodilation, myocardial infarction) or certain surgical procedures (e.g., cardiopulmonary bypass surgery, vascular surgery, renal transplantation) (2). RIR is particularly damaging to tubular epithelial cells (TECs) due to their high energy and oxygen demands (3). The injured TECs release damage-associated molecular patterns (DAMPs), which recruit circulating immune cells and activate renal parenchymal and stromal cells (4).

We have discovered that extracellular cold-inducible RNA-binding protein (eCIRP) is a critical DAMP released after RIR that aggravates AKI, worsens AKI-associated mortality, and increases AKI progression to renal fibrosis (5–9). The specific mechanisms by which eCIRP worsens AKI, however, are not yet well understood. One possibility is that eCIRP augments renal inflammation via its effects on macrophages, the most common type of leukocyte in the kidney (10). We have shown that eCIRP activates macrophage inflammatory responses via TLR4 (11, 12), and activated macrophages have been reported to express natural killer group 2D (NKG2D) membrane receptors (13). Also known as KLRK1, NKG2D is a C-type lectin-like type II transmembrane activating immune receptor primarily expressed by lymphoid cells such as natural killer (NK), NKT, and CD8 T cells (14). NKG2D plays a crucial role promoting innate and adaptive immune system responsiveness to extracellular mediators and stimulating the production of cytokines and cytotoxic molecules, particularly when engaged with its stress induced, membrane-bound ligands (15). In humans, NKG2D ligands consist of ULBP protein family (ULBP1-6), and MHC class I chain-related proteins A and B (MICA and MICB), which are homologous to murine UL16-binding protein-like transcript 1 (MULT-1, homologue of ULBP1), murine RAE-1 family members, and murine MHC class I H60 proteins (16). Normally expressed at very low levels, cell surface expression of NKG2D ligands is markedly upregulated by most cells in response to stressors, including renal tubular epithelial cells upon TLR4 activation (17). Interestingly, NKG2D has been implicated in the pathogenesis of ischemia/reperfusion injury and resulting inflammation in the brain, skin, and lungs (18–21), as well as in kidney transplant rejection (22, 23). Therefore, we hypothesized that eCIRP aggravates renal inflammation following RIR by inducing surface expression of NKG2D on kidney macrophages and of NKG2D ligands on renal tubular epithelial cells. In the present study, we evaluate how the kidney expression of NKG2D and of its ligands change in mice subjected to RIR, the role of eCIRP on the upregulation of this receptor and its ligands, and the consequential effects on the release of proinflammatory cytokines.

## 2 Materials and methods

### 2.1 Reagents

Recombinant murine CIRP (eCIRP) was prepared in-house, as previously described (5). Cell culture media and Pierce protease inhibitor mini tablets (Catalog No: A32953) were purchased from Thermo Fisher Scientific (Carlsbad, CA). The antibodies for Western blotting and immunohistochemistry staining included anti-mouse NKG2D/CD314 (Catalog No: MAB1547) was purchased from R&D Systems (Minneapolis, MN). ULBP-1/MULT-1 polyclonal antibody (Catalog No: 17715-1-AP) was obtained from Proteintech (Rosemont, IL). The anti-β-actin monoclonal antibody (clone AC-15, catalog no: A5441) was obtained from Millipore-Sigma (St. Louis, MO). The anti-mouse PE-NKG2D/CD314 (Catalog No:130207), anti-mouse APC-F4/80 (Catalog No:123116), and NKG2D rat IgG2 antibodies for flow cytometry were purchased from BioLegend (San Diego, CA). Mouse TNFα and IL-6 ELISA kits (catalog No: 558534, 555240) and red blood cell (RBC) lysing buffer (catalog No: 555899) were obtained from BD Biosciences (San Jose, CA).

### 2.2 Animal subjects

Eight- to ten-week-old naïve C57BL/6 mice were purchased from Charles River Laboratories (Wilmington, MA). Age-matched CIRP^−/−^ mice on a C57BL/6 genetic background were generously provided by Dr. Jun Fujita (Kyoto University, Kyoto, Japan). Mice were housed in a temperature-controlled room on a 12 h light cycle and fed a standard Purina rodent chow diet. They were allowed to acclimate to the environment for at least seven days before being used in experiments. All experiments were conducted in accordance with the National Institutes of Health guidelines for the use of experimental animals and were approved by the Institutional Animal Care and Use Committee (IACUC) of the Feinstein Institutes for Medical Research.

### 2.3 Animal model of RIR

RIR injury was induced in mice as described previously (24). Briefly, animals were anesthetized with 2% isoflurane, and their abdomens were shaved and disinfected with 70% isopropyl alcohol followed by 10% povidone-iodine. A 1.5-cm midline incision was made, and the bowel was gently displaced to expose the bilateral renal hila. Microvascular clips were applied to each renal pedicle for 30 minutes. After removing the clips, the abdomen was closed in layers using a continuous 4–0 silk suture. Resuscitation was provided with 0.5 ml of normal saline, and a single subcutaneous dose of buprenorphine (0.05 mg/kg in 100 µl of normal saline) was administered for analgesia immediately following the RIR surgery. At 20 hours post-surgery, mice were euthanized in accordance with the Guide and NIH guidelines using CO_2_ inhalation with a displacement rate of 30% of the chamber volume/min. Blood and renal tissue samples were then collected for subsequent analysis.

### 2.4 Isolation of mouse RTECs and cell culture

Renal tubular epithelial cells (RTECs) were isolated as previously described (25). Briefly, naïve adult C57BL/6 mice were euthanized by CO_2_ inhalation and transcardially perfused with 30 mL of PBS, followed by 20 mL of type II collagenase in PBS (1.3 mg/mL). To minimize contamination, the collagenase solution was filtered through a 0.22 µm sterile syringe filter prior to perfusion. Both kidneys were then harvested, and the capsules and medulla were carefully excised under a surgical microscope. The renal cortex was minced and digested with 1.3 mg/mL collagenase II for 10 minutes at 37 °C. Afterward, undigested tissue was filtered through a 40 µm strainer, and the digestion was halted by adding 20 mL of Dulbecco’s modified Eagle’s medium (DMEM) supplemented with 10% heat-inactivated FBS. The cell suspension then was centrifuged at 50 × g for 10 minutes to collect a pellet enriched with tubular cells. This cell pellet was resuspended in Dulbecco’s modified Eagle’s medium (DMEM) supplemented with 10% heat-inactivated FBS, 2 mM glutamine, and 100 IU/ml penicillin-streptomycin (complete DMEM) and seeded onto a 10 cm collagen-coated culture dish. After three days in culture, RTECs were detached with 0.05% trypsin and seeded into 6- (1.5x10^5^ cells/well) or 96-well (5x10^3^ cells/well) plates for subsequent experiments. We did not use a specialized culture medium for culturing RTECs in this study. To maintain the RTEC phenotype, only first-passage RTECs isolated from the renal cortex were used in the experiments. The kidney resident macrophage protein F4/80 was undetectable in the isolated RTECs. Indeed, this protocol is known to yield a mix of proximal and distal tubular cells with predominance of proximal tubular cells (26).

### 2.5 Isolation and culture of primary mouse peritoneal macrophages

Naïve adult C57BL/6 mice were used to isolate primary mouse peritoneal macrophages (27). Briefly, the mice were euthanized using CO_2_ inhalation, and peritoneal cells were collected by washing the peritoneal cavity with ice-cold PBS containing 2% FBS and 100 IU/ml penicillin-streptomycin. The lavage fluid was centrifuged at 350 × g for 10 minutes at 4 °C. The supernatant was discarded, and the cell pellet was resuspended and cultured in RPMI 1640 medium supplemented with 10% heat-inactivated FBS, 2 mM glutamine, and 100 IU/ml penicillin-streptomycin in 10 cm plates. After 3 hours of incubation at 37 °C in 5% CO_2_, non-adherent cells were gently washed away with warm PBS three times. The remaining adherent cells, comprising more than 90% macrophages, were detached from the plate using a cell scraper, and counted using a hemocytometer. The primary mouse peritoneal macrophages were then cultured alone or co-cultured with RTECs for subsequent experiments.

### 2.6 Co-culture of RTECs and peritoneal macrophages

Co-cultures were established by adding primary mouse peritoneal macrophages to attached primary RTECs. Briefly, primary RTECs isolated from normal kidneys were seeded into 6-well plates (1.5 × 10^5^ cells/well) or 96-well plates (5 × 10³ cells/well) in complete DMEM medium. The RTECs were allowed to adhere at 37 °C in 5% CO_2_. Six hours later, an equal number of peritoneal macrophages was added to the plates with attached RTECs and incubated overnight. The culture medium was then replaced with Opti-MEM reduced-serum medium (Thermo Fisher Scientific).

### 2.7 Stimulation of RTECs and peritoneal macrophages with eCIRP and anti-NKG2D neutralizing antibody

RTECs were cultured overnight in complete DMEM and peritoneal macrophages in complete RPMI medium. The media were then replaced with Opti-MEM medium 1 hour prior to treatment. Recombinant mouse CIRP (eCIRP) was prepared in-house and validated with quality control assays as previously described (5). For the NKG2D flow cytometry study, peritoneal macrophages were treated with varying doses of eCIRP (0, 0.5, 1, and 2 µg/mL) for 16 hours. For the MULT-1 Western blotting, cells were treated with 1 µg/mL of eCIRP for 16 hours. In co-culture experiments, RTECs and peritoneal macrophages were incubated with 1 µg/mL eCIRP, with or without 2 µg/mL NKG2D neutralizing antibody. After 16 hours of co-culture, 6-well plate cell lysates were harvested for Western blotting, and 96-well plate supernatants were collected and centrifuged at 350 × g for 10 minutes for proinflammatory cytokine analysis.

### 2.8 Flow cytometry

To evaluate changes in the NKG2D expression of kidney resident macrophages, F4/80^+^ cells were analyzed for NKG2D using flow cytometry. Kidneys of WT or CIRP^−/−^ mice subjected to RIR were minced and digested with 1.3 µg/mL collagenase II and 0.7 µg/mL collagenase I for 30 minutes at 37 °C. The processed samples were then passed through a 40-μm strainer and centrifuged at 50 × g for 10 minutes. The single cell pellet was then resuspended, depleted of red blood cells (RBCs) using RBC lysis buffer, and washed with FACS buffer. Cell surface expression of the NKG2D was assessed using PE-conjugated anti-mouse NKG2D and APC-conjugated anti-mouse F4/80 antibodies and rat IgG2 isotype control. After incubation for 60 minutes at 4 °C, cells were washed in FACS buffer and fixed in 2% (w/v) paraformaldehyde (Sigma-Aldrich, St. Louis, MO). Unstained cells were used to calibrate the flow cytometer voltage settings. Acquisition was performed at 20,000 events using a BD LSR Fortessa flow cytometer (BD Biosciences), and data were analyzed with FlowJo software (Tree Star, Ashland, OR).

### 2.9 Western blotting assay

Kidney fragments were collected, snap frozen, crushed, and resuspended in lysis buffer (10 mM Tris-HCl at pH 7.5, 100 mM sodium chloride, 1 mM EGTA, 1 mM EDTA, 1 mM sodium orthovanadate, Triton X-100) containing Pierce protein inhibitor (1 mini tablet/10 mL) for 15 minutes at 4 °C. The suspension was sonicated and then centrifuged at 10,000 × g for 10 minutes. The supernatant was collected, measured protein concentration and mixed with 2 × protein loading buffer containing 2-mercaptoethanol and boiled for 5 minutes before loading onto SDS-PAGE. Cell lysates were fractionated on 4%-12% Bis-Tris gels. After electrophoresis, proteins were transferred onto nitrocellulose membranes, after blocking with 0.1% casein in Tris-buffered saline, the membranes were incubated with rabbit anti-MULT-1 antibody (1:500), rabbit anti-mouse NKG2D antibody (1:1000) and anti-mouse β-actin antibody overnight at 4 °C. Membranes were washed with PBST (PBS at pH 7.4 containing 0.05% Tween-20). The target bands were visualized using infrared dye-labeled secondary Abs and Odyssey Clx image system (Li-Cor Biosciences). The immunostaining bands for NKG2D and MULT-1 were scored using ImageJ software to determine the semiquantitative immunoreactive score.

### 2.10 Enzyme-linked immunosorbent assay

To analyze cytokine secretion by RTECs and peritoneal macrophages, medium supernatants were collected from 96-well plates at 16 h after treatment of eCIRP (1µg/ml) with or without NKG2D neutralizing antibody (2 µg/ml). TNFα and IL-6 in the culture supernatants were measured using mouse-specific ELISA from kits from BD Biosciences according to the manufacturer’s instructions.

### 2.11 Immunohistochemistry staining and assessment

Kidney samples from WT and CIRP^−/−^ mice subjected to RIR were fixed with formalin, embedded in paraffin, and microsectioned into 5-µm slices. The sections were incubated overnight at 4 °C with rabbit anti-mouse MULT-1 antibody (1:50) or rabbit anti-mouse NKG2D antibody (1:50). Following this, the sections were incubated with HRP-labeled goat anti-rabbit secondary antibody (Catalog No: 31402, Thermo Fisher Scientific) and visualized by staining with 3,3′-diaminobenzidine (Catalog No: DB801, Biocare Medical, Pacheco, CA). The immunostaining for MULT-1 and NKG2D was assessed using ImageJ software to determine the semiquantitative immunoreactive score.

### 2.12 Statistical analysis

Data are expressed as mean ± SEM. Data analyses were performed using GraphPad Prism software (GraphPad Software, San Diego, CA). The Student’s *t-*Test was applied for comparisons between two groups; one-way ANOVA was used for comparisons among multiple groups. A *p*-value ≤ 0.05 was considered statistically significant.

## 3 Results

### 3.1 RIR upregulates renal expression of MULT-1 and NKG2D, and renal macrophage expression of NKG2D

**Figure 1:**
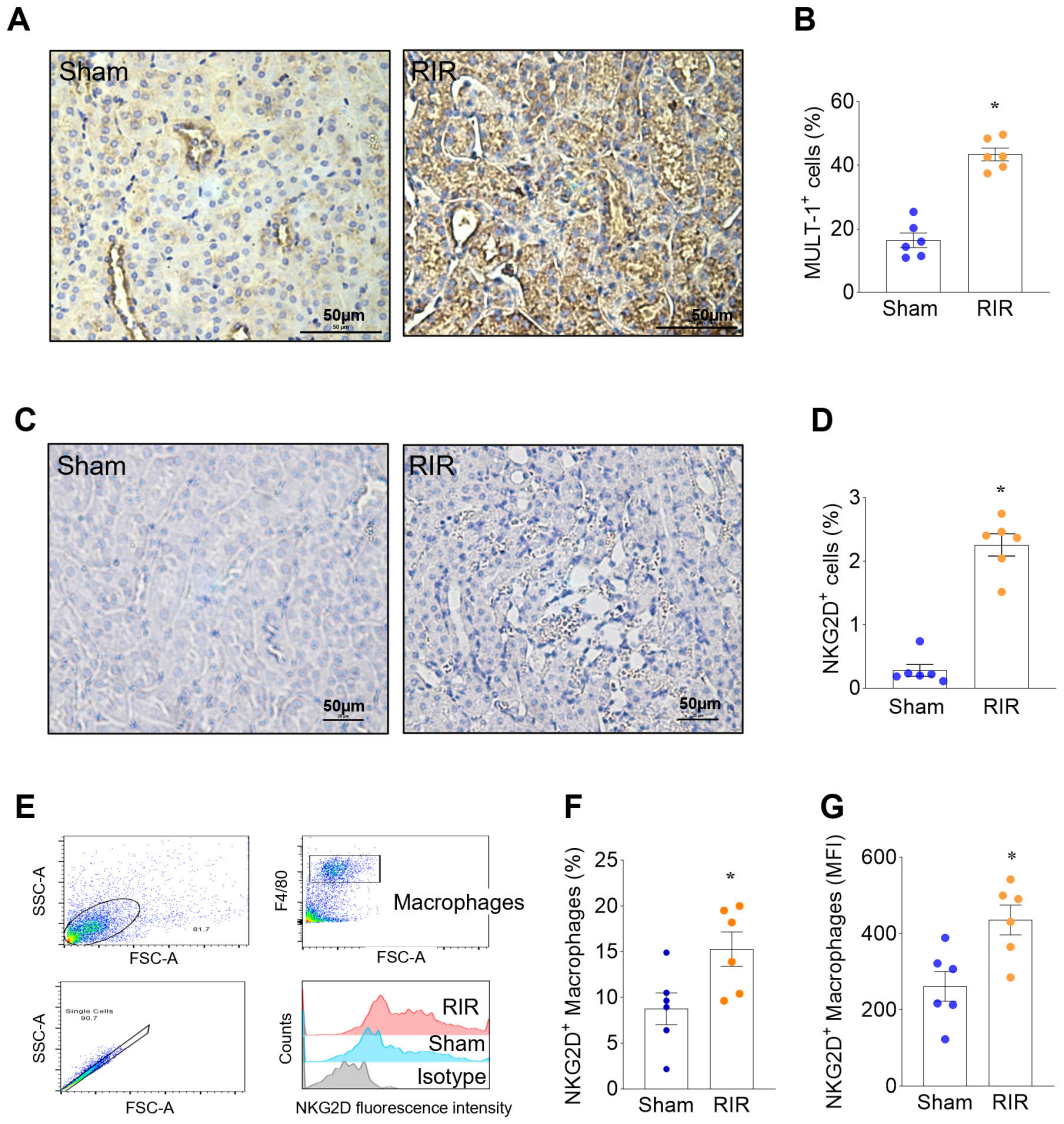
RIR upregulates renal expression of MULT-1 and renal macrophage expression of NKG2D. **(A, B)** The kidneys of mice collected 20 h after RIR showed a significant increase in the expression of MULT-1 compared with those of sham. **(C, D)** Similarly, the expression of NKG2D was minimal in the kidneys of sham mice, but it was significantly upregulated in the kidneys of RIR mice. Immunohistochemistry (IHC); representative images. The percentage of positive (brown) cells was quantified using ImageJ, n=6/group; mean ± standard error (SEM); t-Test, *p<0.05 vs. Sham.**(E)** Single cell suspensions from the kidneys of sham and RIR mice were stained with F4/80 NKG2D antibodies and analyzed by flow cytometry. **(F)** The percentage of NKG2D^+^ macrophages and **(G)** the mean fluorescence intensity (MFI) of NKG2D were significantly increased in renal macrophages of Sham and RIR mice. Flow cytometry; n=6/group; mean ± SEM; Student’s t-Test, *p<0.05 vs. Sham..

To investigate changes in the expression of MULT-1 and NKG2D during AKI, we subjected WT-mice to RIR injury. 20 hours later, the kidneys of mice subjected to RIR showed a significant 2.75-fold increase in the expression of MULT-1 compared with sham, as measured by IHC (**Figures 1A, B**). Similarly, the expression of NKG2D was minimal in the sham group, significantly upregulated by 8.07-fold in the kidneys of RIR mice (**Figures 1C, D**). These results indicate that RIR causes kidney cells to upregulate the expression of the NKG2D receptor and its ligand MULT-1. To determine whether the NKG2D^+^ cells induced by RIR included macrophages, we assessed co-expression of NKG2D and F4/80 by flow cytometry. At 20 hours after RIR, compared with sham, there was a significant 74.4% increase in the proportion of renal macrophages expressing NKG2D, and a significant 66.4% increase in the mean fluorescence intensity (MFI) of NKG2D^+^ macrophages (**Figures 1E–G**). Therefore, RIR markedly upregulates renal macrophage surface expression of NKG2D receptors.

### 3.2 RIR induces NKG2D^+^ renal macrophages via eCIRP

**Figure 2:**
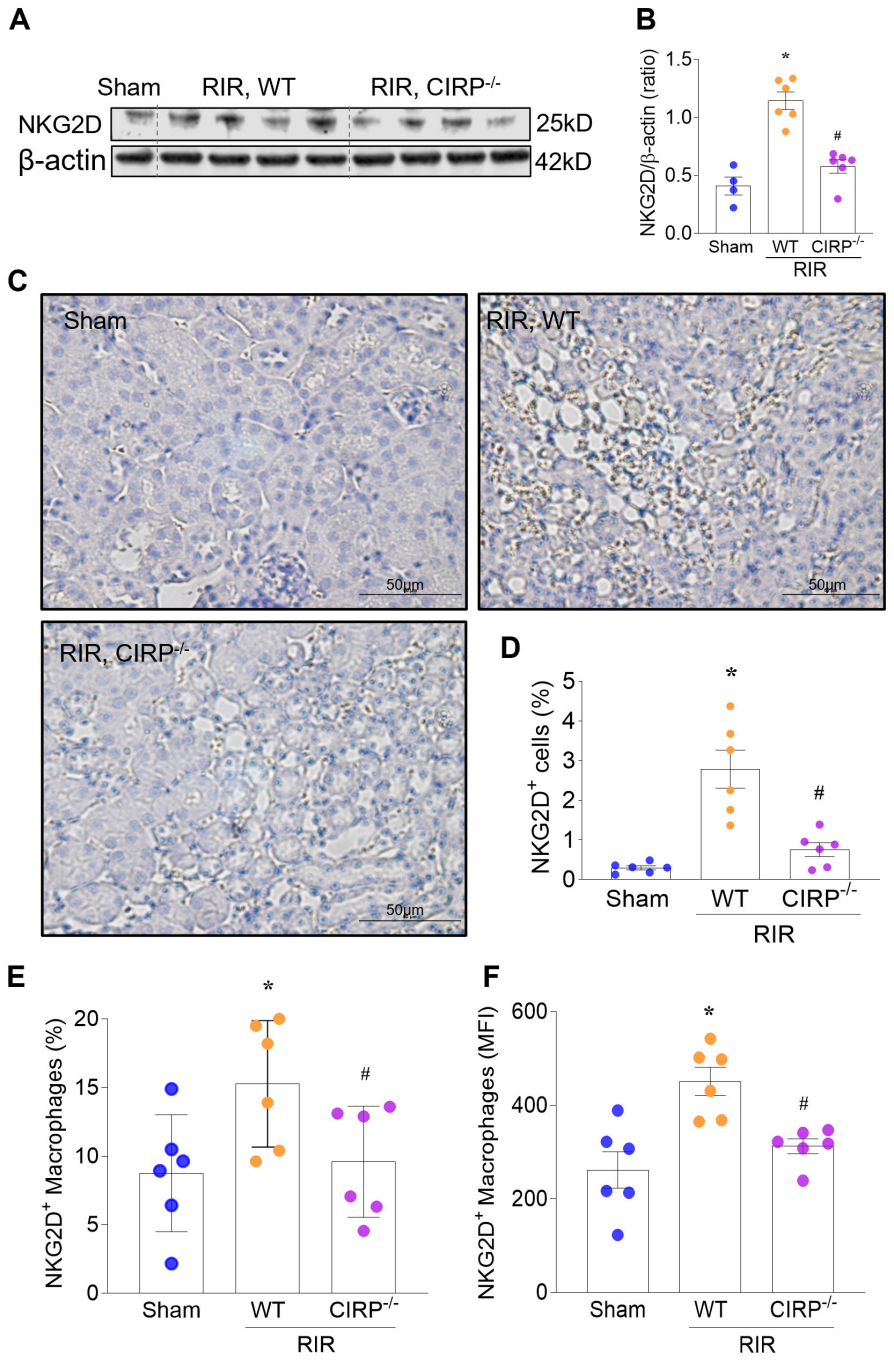
RIR induces NKG2D+ renal macrophages via eCIRP. We subjected WT and CIRP^−/−^ mice to RIR. **(A, B)** At 20 h after surgery, the total renal NKG2D protein significantly increased in WT mice subjected to RIR compared with sham. However, the RIR-induced increase in NKG2D expression was nearly completely abrogated in CIRP^−/−^ mice. Western blotting, ImageJ quantification; β-actin, internal control, n=4-6/group; mean ± standard error (SEM); ANOVA, *p<0.05 vs. Sham, ^#^p<0.05 vs. RIR WT mice. **(C, D)** Compared with sham, the NKG2D protein expression was also significantly upregulated in kidney sections of RIR WT mice, but only minimally in kidney sections of RIR CIRP^−/−^ mice. IHC; representative images. The percentage of positive (brown) cells was quantified using ImageJ, n=6/group; mean ± SEM; ANOVA, *p<0.05 vs. Sham, ^#^p<0.05 vs. RIR WT mice. **(E, F)** Compared with sham, RIR significantly induced renal NKG2DR^+^ macrophage expression in WT, but not in CIRP^−/−^ mice. Flow cytometry; n=6/group; mean ± SEM; mean ± SEM; ANOVA, *p<0.05 vs. Sham, ^#^p<0.05 vs. RIR WT mice.

RIR induces kidney expression and release of eCIRP, and eCIRP can activate macrophages (5, 9, 11). Thus, given the observed increase in expression of NKG2D in the kidneys of RIR mice, we hypothesized that the increased levels of eCIRP explain the increased expression of NKG2D in renal macrophages. To investigate this, we assessed the RIR-induced renal expression of NKG2D in WT and CIRP^−/−^ mice by Western blotting. At 20 h after surgery, the total renal NKG2D protein increased 2.85-fold in WT mice subjected to RIR compared with sham (**Figures 2A, B**). However, the RIR-induced increase in NKG2D expression was nearly completely abrogated in CIRP^−/−^ mice. Consistent with these results, IHC staining showed a 9.5-fold increase in NKG2D expression in RIR kidney sections of WT, but only minimal staining in sections of CIRP^−/−^ mice (**Figures 2C, D**). These studies show that deficiency in CIRP impedes the increase in overall renal expression of NKG2D after RIR. We next evaluated eCIRP in RIR-induction of NKG2D on renal macrophages using flow cytometry. Compared with sham, RIR significantly induced renal NKG2DR^+^ macrophage expression in WT, but not in CIRP^−/−^ mice (**Figures 2E, F**). Taken together, these studies show that eCIRP plays a key role in upregulating NKG2D expression in renal macrophages following RIR.

### 3.3 eCIRP directly activates NKG2D expression in macrophages

**Figure 3:**
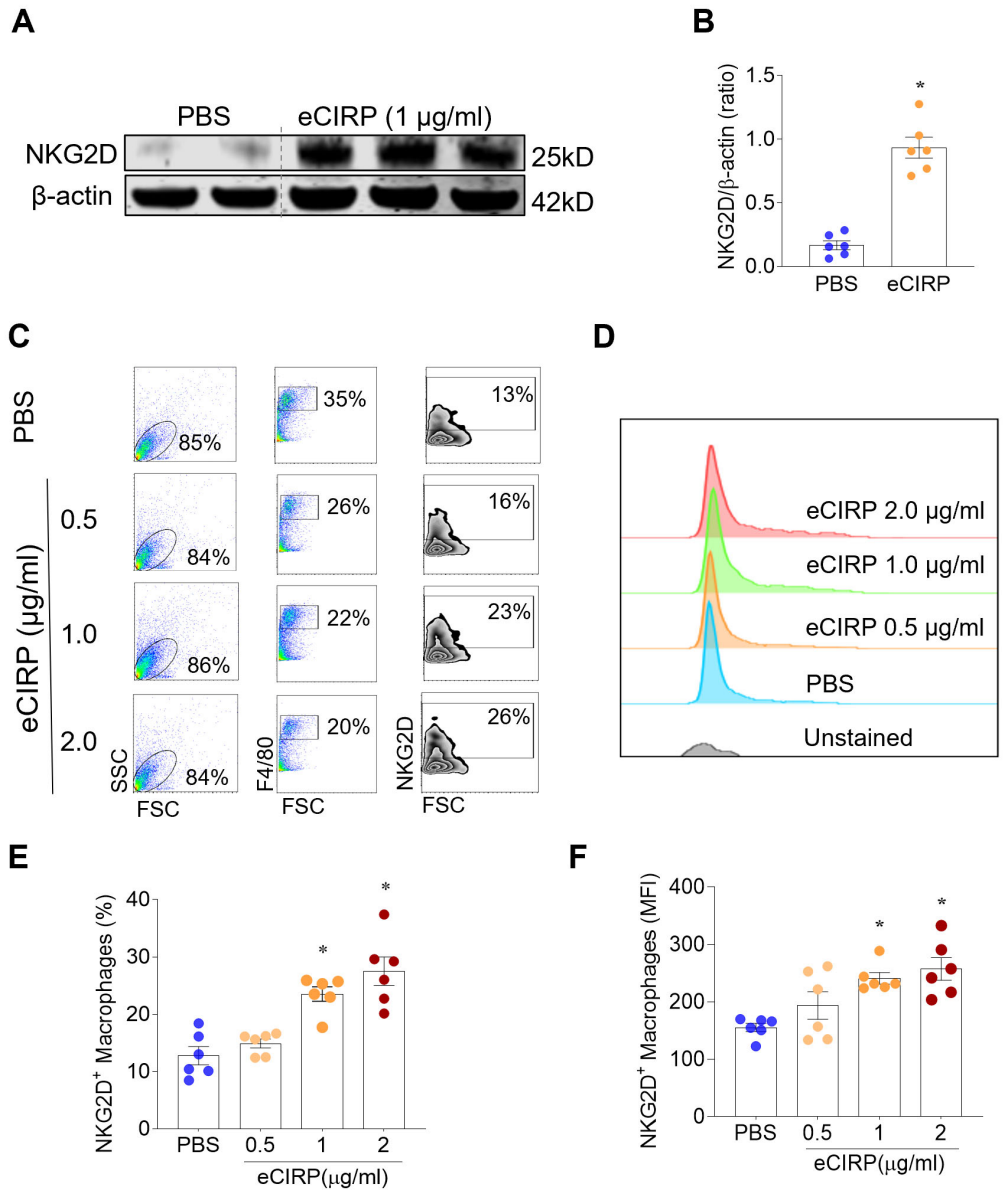
eCIRP directly activates NKG2D expression in peritoneal macrophages. Peritoneal macrophages from naïve WT mice were stimulated with recombinant mouse CIRP (eCIRP; 1 µg/mL) for 16 h. **(A, B)***In vitro* stimulation with eCIRP caused a pronounced and significant increase in NKG2D protein in the macrophage lysates. Western blotting; β-actin, internal control; ImageJ quantification; n=6/group; mean ± standard error (SEM); Student’s t-Test, *p<0.05. **(C, D)** Peritoneal macrophages from WT mice were plated in 6-well culture plates overnight. The cells were then stimulated by 0, 0.5, 1 or 2 µg/ml of eCIRP, followed by the detection of NKG2D by flow cytometry. eCIRP stimulation dose-dependently increased NKG2D surface expression of peritoneal macrophages, as indicated by both **(E)** the percentage of positive cells and **(F)** the MFI of NKG2D in the cultured macrophages. Flow cytometry; n=6/group; mean ± SEM; ANOVA, *p<0.05 vs. PBS.

CIRP^−/−^ mice are also deficient in intracellular CIRP. Thus, to exclude possible effects of intracellular CIRP, we collected peritoneal macrophages from naïve WT mice and stimulated with recombinant mouse CIRP (eCIRP; 1 µg/mL) overnight (5, 28). *In vitro* stimulation with eCIRP caused a significant 4.6-fold increase in NKG2D protein in the macrophage lysates (**Figures 3A, B**). We also assessed the effects of eCIRP on the surface expression of NKG2D by flow cytometry (**Figures 3C, D**). Direct stimulation with eCIRP dose-dependently increased NKG2D surface expression of peritoneal macrophages, as indicated by both the percentage of positive cells and mean fluorescence intensity (MFI) (**Figures 3E, F**). We further confirmed these findings in cultured primary bone marrow-derived macrophages (**Supplementary Figure 1**). These results indicate that eCIRP directly promotes the expression of the NKG2D receptor in renal macrophages.

### 3.4 eCIRP upregulates MULT-1 in renal tissues and in RTECs after RIR

**Figure 4:**
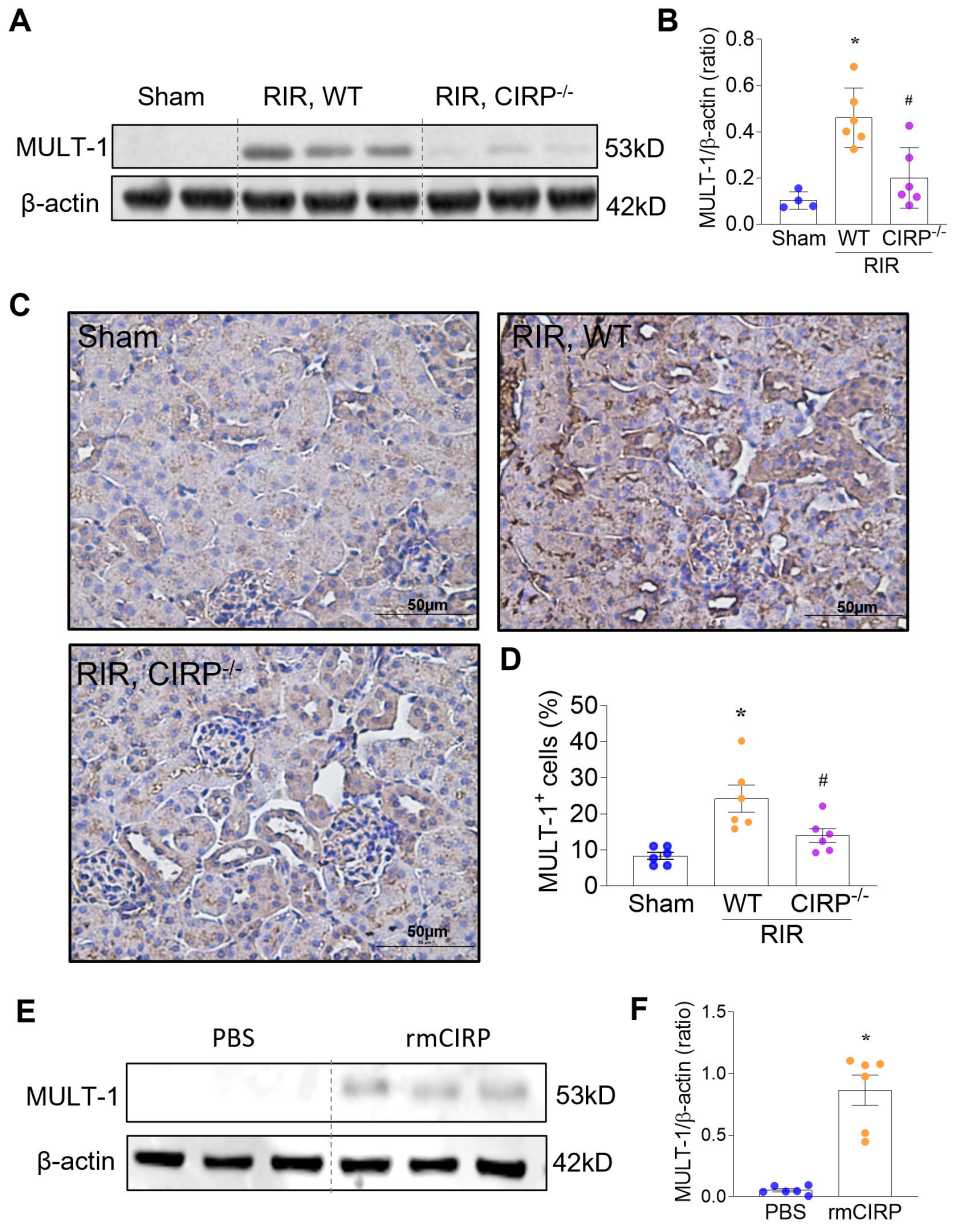
eCIRP upregulates MULT-1 in RTECs after RIR. We subjected WT and CIRP^−/−^ mice to RIR. **(A, B)** At 20 h after surgery, the total renal MULT-1 protein significantly increased in WT mice subjected to RIR compared with sham. However, the RIR-induced increase in MULT-1 expression was significantly diminished in CIRP^−/−^ mice. Western blotting, ImageJ quantification; β-actin, internal control, n=4-6/group; mean ± standard error (SEM); ANOVA, *p<0.05 vs. Sham, ^#^p<0.05 vs. RIR WT mice. **(C, D)** Compared with sham, MULT-1 protein expression was also significantly upregulated in kidney sections of RIR WT mice, but it was significantly reduced in kidney sections of RIR CIRP^−/−^ mice. IHC; representative images. The percentage of positive (brown) cells was quantified using ImageJ, n=6/group; mean ± SEM; ANOVA, *p<0.05 vs. Sham, ^#^p<0.05 vs. RIR WT mice. **(E, F)** To examine whether eCIRP has a direct effect on renal cells, primary RTECs from naïve adult mice were isolated and treated them with recombinant mouse CIRP (eCIRP,1 µg/ml) for 16 hours. Stimulation with eCIRP significantly upregulated RTEC expression of MULT-1. Western blotting, ImageJ quantification; β-actin, internal control, n=6/group; mean ± standard error (SEM); ANOVA, *p<0.05 vs. Sham, ^#^p<0.05 vs. RIR WT mice.

The immune receptor NKG2D is activated via its binding to membrane-bound ligands on the surface of target cells. Observing eCIRP’s involvement in NKG2D expression in macrophages led us to consider its potential impact on NKG2D ligands on tubular epithelial cells. Since we showed that MULT-1 is markedly upregulated in RTECs after RIR, we investigated whether eCIRP regulates RTEC expression of MULT-1. At 20 h after surgery, the total renal MULT-1 protein significantly increased by 4.6-fold in WT mice subjected to RIR compared with sham. However, the RIR-induced increase in total renal MULT-1 was significantly diminished by 56.5% in CIRP^−/−^ mice (Western blotting; **Figures 4A, B**). Similarly, compared with sham, MULT-1 protein expression was also significantly upregulated by 2.9-fold in kidney sections of RIR WT mice, but it was significantly reduced by 40.8% in kidney sections of RIR CIRP^−/−^ mice (IHC; **Figures 4C, D**). We also observed a significant reduction in the expression of RAE-1, another ligand of NKG2D, in RIR kidneys of CIRP^−/−^ mice compared to those of WT mice (**Supplementary Figures 2A, B**). To examine whether eCIRP has a direct effect on renal cells, we isolated primary RTECs from naïve adult mice and treated them with recombinant mouse CIRP (eCIRP,1 µg/ml). Stimulation with eCIRP significantly upregulated RTEC expression of MULT-1 by 16.3-fold relative to control (PBS-treated) RTECs (**Figures 4E, F**). Stimulation with eCIRP also increased RAE-1 expression in RTECs (**Supplementary Figures 2C, D**). Taken together, these findings demonstrate that the increased expression of NKG2D ligands in RTECs after RIR is mediated by eCIRP.

### 3.5 eCIRP induces the expression of proinflammatory cytokines via the NKG2D axis

**Figure 5:**
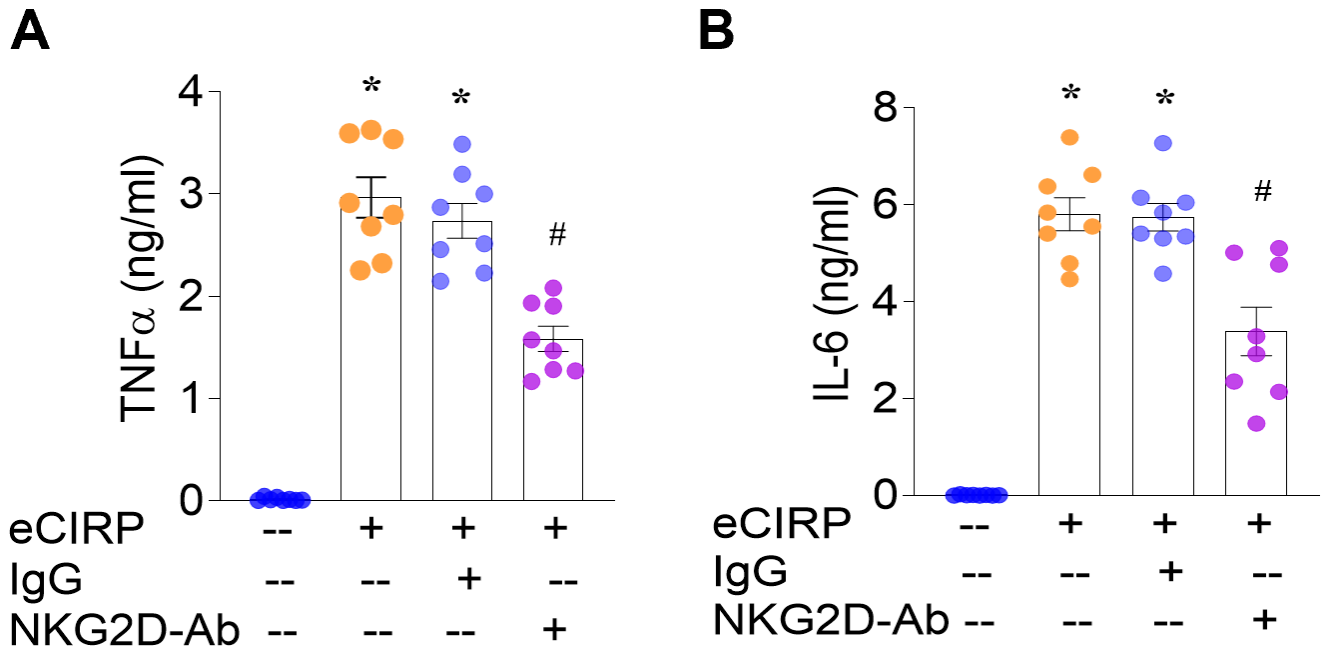
eCIRP induces the expression of proinflammatory cytokines via the NKG2D axis. Naïve WT RTEC and peritoneal macrophage co-cultures were stimulated with 1 µg/ml eCIRP in the presence or absence of anti-NKG2D neutralizing antibody (Ab) or IgG control (2 µg/ml). **(A, B)** 16 hours later, supernatant levels of TNFα and IL-6 were equally elevated in co-cultures stimulated with eCIRP and eCIRP+IgG. However, NKG2D-neutralizing Ab significantly reduced the eCIRP-induced release of TNFα and IL-6, compared to IgG. ELISA; n=8/group; mean ± standard error (SEM); ANOVA, *p<0.05 vs. PBS-treated group, ^#^p<0.05 vs. eCIRP+IgG group.

After determining that eCIRP induces the expression of NKG2D in kidney macrophages and of its ligands MULT-1 and RAE-1 in RTECs, we investigated the biological significance of the interaction between these cells. To investigate whether NKG2D signaling contributes to eCIRP-induced inflammation, we co-cultured RTECs and peritoneal macrophages from naïve mice and stimulated the co-cultured cells with eCIRP in the presence or absence of an anti-NKG2D neutralizing antibody (Ab) or IgG control. We then collected the supernatant from the co-culture medium and found that eCIRP stimulation significantly increased the production of TNFα and IL-6 by 18.3- and 46.0-fold, respectively, compared with controls. As expected, TNFα and IL-6 levels were equally elevated in co-cultures stimulated with eCIRP and eCIRP+IgG. However, NKG2D-neutralizing Ab significantly reduced the eCIRP-induced release of TNFα and IL-6 by 42% and 41%, respectively, compared to IgG (**Figures 5A, B**). These results demonstrate that eCIRP promotes the release of proinflammatory cytokines, while NKG2D neutralization attenuates this effect in co-cultured macrophages and RTECs. Therefore, eCIRP-induces inflammation, at least in part, by promoting the interaction of NKG2D^+^ renal macrophages and its ligands on RTECs.

## 4 Discussion

Renal ischemia-reperfusion (RIR) injury disrupts the supply of oxygen and nutrients and impairs waste removal, ultimately resulting in cellular damage. We have discovered that injured kidney cells release eCIRP, which then promotes hyperinflammatory responses that further aggravate renal injury, ultimately causing renal dysfunction and progression to renal fibrosis (5–9). The specific mechanisms by which eCIRP aggravates renal injury, however, remain poorly understood. Macrophages are the most common type of leukocyte in the kidney and have been identified as key participants in the pathogenesis of RIR and AKI (10). These cells play essential roles in efferocytosis, phagocytosis, inflammation, and immune regulation by expressing a diverse array of pattern recognition receptors (PRRs) on their surface. These receptors can be activated upon binding to host-derived damage-associated molecular patterns (DAMPs) or microbial-derived pathogen-associated molecular patterns (PAMPs) (29). Indeed, we have discovered that eCIRP is a strong macrophage activator, promoting macrophage release of IL-1β and TNFα and vital METosis (11, 12, 28). Natural killer group 2D (NKG2D) is an activating immune receptor extensively and almost exclusively studied on natural killer (NK) cells and certain T cell subsets (30). Activated macrophages have been reported to express NKG2D (13). Therefore, we hypothesized that eCIRP aggravates renal inflammation following RIR by inducing surface expression of NKG2D on kidney macrophages and of NKG2D ligands on renal tubular epithelial cells.

The prevailing consensus has been that NKG2D receptors are not expressed on macrophages. However, very few studies have evaluated the expression of NKG2D on these cells. Jamieson et al. reported surface expression of NKG2D on bone marrow-derived and peritoneal macrophages following stimulation with LPS, IFN-γ, or IFN-α/β (14). In the present study, we clearly demonstrate that renal macrophages activated by eCIRP can and do express NKG2D. Our studies include not only detection of NKG2D in renal tissue but IHC, but also colocalization with F4/80 by flow cytometry. In the kidney, F4/80 is highly expressed by resident macrophages but it is also expressed by monocyte-derived macrophages. We also showed that peritoneal macrophages stimulated with eCIRP undergo a pronounced and significant increase in NKG2D total protein expression by Western blotting and surface protein expression by flow cytometry. Combined, our results provide robust evidence that eCIRP-activated macrophages express NKG2D.

It is generally believed that activation of the NKG2D on immune cells requires the binding of its specific ligands, which are typically expressed on stressed, virus infected, or transformed cells. In the absence of these ligands, the NKG2D receptor remains inactive. In the present study, we observed that, in the absence of target cells expressing NKG2D ligands, eCIRP is sufficient to induce the expression of NKG2D receptors in peritoneal macrophages. This finding raises intriguing questions about the mechanisms by which DAMPs regulate NKG2D receptor expression. Previous studies have demonstrated that eCIRP-induced inflammation occurs through its binding to the TLR4-myeloid differentiation factor 2 (MD2) receptor complex, TREM-1 or IL-6 receptor on macrophages, thereby regulating the inflammatory response (5, 31, 32). It is well established that the NKG2D receptor itself is not regulated by TLR4 receptors, and no evidence has been reported suggesting regulation of the NKG2D receptor by TREM-1 or IL-6 receptors. Therefore, exactly how eCIRP regulates the expression of NKG2D in macrophages remains to be elucidated. However, unlike NKG2D, NKG2D ligands can be regulated by the TLR4 receptor through multiple downstream mechanisms (33). There is a report indicating that activated macrophages in C57BL/6 mice can express the NKG2D ligand RAE-1, though not H60 or MULT-1 (34). It is possible that eCIRP may induce NKG2D receptor expression indirectly by upregulating the ligand RAE-1 in macrophages through activation of the TLR4 pathway. However, this hypothesis does not explain why eCIRP downregulated the NKG2D ligand MULT-1 and decreased cytokine release when eCIRP-induced NKG2D receptor expression was blocked using a neutralizing antibody, as MULT-1 has not been shown to be expressed in macrophages of C57BL/6 mice. IFN-α/β have been shown to upregulate macrophage expression of NKG2D (14). We have shown, using peritoneal macrophages, that eCIRP binding to TLR4 leads to activation the STING pathway – via both the MyD88 and TRIFF pathways – resulting in the upregulation and release of IFN-α/β (12). Thus, it is possible that eCIRP regulates NKG2D expression via autocrine stimulation with type I interferons. The precise mechanism by which eCIRP induces NKG2D receptor expression remains unexamined and warrants further in-depth investigation.

Proinflammatory cytokines such as TNF-α and IL-6 play a key role in promoting the inflammatory response following RIR (35). eCIRP, as a strong stress-induced DAMP, has been shown to induce the release of pro-inflammatory cytokines, including TNF-α and IL-6, in bone marrow-derived macrophages, tissue-resident macrophages, and various macrophage cell lines (36–38). The interaction of NKG2D and its ligands has been identified to be crucial molecular pathway in inflammation and organ dysfunction (39–41). To explore whether the NKG2D axis is involved in eCIRP-induced production of proinflammatory cytokines, we co-cultured peritoneal macrophages with RTECs and stimulated them with eCIRP in the presence or absence of an NKG2D-neutralizing antibody. Our results demonstrated that stimulation with eCIRP significantly increased the production of TNF-α and IL-6. Importantly, NKG2D neutralization significantly reduced this cytokine release. These findings indicate that the NKG2D axis is an important contributor to eCIRP-induced release of proinflammatory cytokines, highlighting the potential involvement of the NKG2D axis in the pathogenesis of RIR.

In summary, our *in vivo* studies demonstrate that eCIRP promotes the expression of NKG2D and of its ligand MULT-1 in renal injury induced by RIR. Notably, we provide the first evidence that eCIRP upregulates NKG2D receptor expression in macrophages independently of its ligands. Furthermore, neutralization of NKG2D resulted in a marked reduction of proinflammatory cytokines in eCIRP-stimulated macrophage and RTEC co-cultures. In conclusion, we have discovered a critical role for the NKG2D axis in eCIRP-mediated activation of macrophages in the kidney following RIR. Additional studies are needed to establish the pathophysiological role of NKG2D^+^ macrophages and the possible therapeutic value of targeting these cells in patients with AKI.

## Data Availability

The raw data supporting the conclusions of this article will be made available by the authors, without undue reservation.

## References

[1] Ostermann M Lumlertgul N Jeong R See E Joannidis M James M . Acute kidney injury. Lancet. (2025) 405:241–56. doi: 10.1016/S0140-6736(24)02385-7, PMID: 39826969

[2] Turgut F Awad AS Abdel-Rahman EM . Acute kidney injury: Medical causes and pathogenesis. J Clin Med. (2023) 12:375. doi: 10.3390/jcm12010375, PMID: 36615175 PMC9821234

[3] Li Z Lu S Li X . The role of metabolic reprogramming in tubular epithelial cells during the progression of acute kidney injury. Cell Mol Life Sci. (2021) 78:5731–41. doi: 10.1007/s00018-021-03892-w, PMID: 34185125 PMC11073237

[4] Tang PM Nikolic-Paterson DJ Lan HY . Macrophages: versatile players in renal inflammation and fibrosis. Nat Rev Nephrol. (2019) 15:144–58. doi: 10.1038/s41581-019-0110-2, PMID: 30692665

[5] Qiang X Yang WL Wu R Zhou M Jacob A Dong W . Cold-inducible RNA-binding protein (CIRP) triggers inflammatory responses in hemorrhagic shock and sepsis. Nat Med. (2013) 19:1489–95. doi: 10.1038/nm.3368, PMID: 24097189 PMC3826915

[6] Zhang F Hu Z Jacob A Brenner M Wang P . An eCIRP inhibitor attenuates fibrosis and ferroptosis in ischemia and reperfusion induced chronic kidney disease. Mol Med. (2025) 31:11. doi: 10.1186/s10020-025-01071-2, PMID: 39794717 PMC11724597

[7] Siskind S Zhang F Brenner M Wang P . Extracellular CIRP induces acute kidney injury via endothelial TREM-1. Front Physiol. (2022) 13:954815. doi: 10.3389/fphys.2022.954815, PMID: 36246143 PMC9558214

[8] Siskind S Royster W Brenner M Wang P . A novel eCIRP/TREM-1 pathway inhibitor attenuates acute kidney injury. Surgery. (2022) 172:639–47. doi: 10.1016/j.surg.2022.02.003, PMID: 35292178 PMC9283225

[9] Cen C Yang WL Yen HT Nicastro JM Coppa GF Wang P . Deficiency of cold-inducible ribonucleic acid-binding protein reduces renal injury after ischemia-reperfusion. Surgery. (2016) 160:473–83. doi: 10.1016/j.surg.2016.04.014, PMID: 27267546 PMC4938765

[10] Melkonian AL Cheung MD Erman EN Moore KH Lever JMP Jiang Y . Single-cell RNA sequencing and spatial transcriptomics reveal unique subpopulations of infiltrating macrophages and dendritic cells following AKI. Am J Physiol Renal Physiol. (2025) 328:F907–20. doi: 10.1152/ajprenal.00059.2025, PMID: 40331777

[11] Li Z Fan EK Liu J Scott MJ Li Y Li S . Cold-inducible RNA-binding protein through TLR4 signaling induces mitochondrial DNA fragmentation and regulates macrophage cell death after trauma. Cell Death Dis. (2017) 8:e2775. doi: 10.1038/cddis.2017.187, PMID: 28492546 PMC5584526

[12] Chen K Cagliani J Aziz M Tan C Brenner M Wang P . Extracellular CIRP activates STING to exacerbate hemorrhagic shock. JCI Insight. (2021) 6:e143715. doi: 10.1172/jci.insight.143715, PMID: 34291735 PMC8410031

[13] Stojanovic A Correia MP Cerwenka A . The NKG2D/NKG2DL axis in the crosstalk between lymphoid and myeloid cells in health and disease. Front Immunol. (2018) 9:827. doi: 10.3389/fimmu.2018.00827, PMID: 29740438 PMC5924773

[14] Jamieson AM Diefenbach A McMahon CW Xiong N Carlyle JR Raulet DH . The role of the NKG2D immunoreceptor in immune cell activation and natural killing. Immunity. (2002) 17:19–29. doi: 10.1016/s1074-7613(02)00333-3, PMID: 12150888

[15] Wensveen FM Jelencic V Polic B . NKG2D: A master regulator of immune cell responsiveness. Front Immunol. (2018) 9:441. doi: 10.3389/fimmu.2018.00441, PMID: 29568297 PMC5852076

[16] Zingoni A Molfetta R Fionda C Soriani A Paolini R Cippitelli M . NKG2D and its ligands: “One for all, all for one. Front Immunol. (2018) 9:476. doi: 10.3389/fimmu.2018.00476, PMID: 29662484 PMC5890157

[17] Chen GE Wu H Ma J Chadban SJ Sharland A . Toll-like receptor 4 engagement contributes to expression of NKG2D ligands by renal tubular epithelial cells. Nephrol Dial Transplant. (2011) 26:3873–81. doi: 10.1093/ndt/gfr234, PMID: 21555390

[18] Liu L Yang Y Wu T Du J Long F . NKG2D knockdown improves hypoxic-ischemic brain damage by inhibiting neuroinflammation in neonatal mice. Sci Rep. (2024) 14:2326. doi: 10.1038/s41598-024-52780-3, PMID: 38282118 PMC10822867

[19] Makita K Otsuka N Tomaru U Taniguchi K Kasahara M . NKG2D ligand expression induced by oxidative stress mitigates cutaneous ischemia-reperfusion injury. J Histochem Cytochem. (2023) 71:61–72. doi: 10.1369/00221554221147582, PMID: 36762536 PMC10088101

[20] David C Ruck T Rolfes L Mencl S Kraft P Schuhmann MK . Impact of NKG2D signaling on natural killer and T-cell function in cerebral ischemia. J Am Heart Assoc. (2023) 12:e029529. doi: 10.1161/JAHA.122.029529, PMID: 37301761 PMC10356034

[21] Calabrese DR Aminian E Mallavia B Liu F Cleary SJ Aguilar OA . Natural killer cells activated through NKG2D mediate lung ischemia-reperfusion injury. J Clin Invest. (2021) 131:e137047. doi: 10.1172/JCI137047, PMID: 33290276 PMC7852842

[22] Diebold M Vietzen H Heinzel A Haindl S Herz CT Mayer K . Natural killer cell functional genetics and donor-specific antibody-triggered microvascular inflammation. Am J Transplant. (2024) 24:743–54. doi: 10.1016/j.ajt.2023.12.005, PMID: 38097018

[23] Zhu L Karakizlis H Weimer R Morath C Ekpoom N Ibrahim EH . Circulating NKG2A-NKG2D+ CD56dimCD16+ natural killer (NK) cells as mediators of functional immunosurveillance in kidney transplant recipients. Ann Transplant. (2020) 25:e925162. doi: 10.12659/AOT.925162, PMID: 33349627 PMC7763919

[24] McGinn J Zhang F Aziz M Yang WL Nicastro J Coppa GF . The protective effect of a short peptide derived from cold-inducible RNA-binding protein in renal ischemia-reperfusion injury. Shock. (2018) 49:269–76. doi: 10.1097/SHK.000000000000098, PMID: 28930914 PMC5809308

[25] Hu Z Zhang F Brenner M Jacob A Wang P . The protective effect of H151, a novel STING inhibitor, in renal ischemia-reperfusion-induced acute kidney injury. Am J Physiol Renal Physiol. (2023) 324:F558–F67. doi: 10.1152/ajprenal.00004.2023, PMID: 37102684 PMC10228668

[26] Ding W Yousefi K Shehadeh LA . Isolation, characterization, and high throughput extracellular flux analysis of mouse primary renal tubular epithelial cells. J Vis Exp. (2018) 20:57718. doi: 10.3791/57718-v, PMID: 29985358 PMC6101965

[27] Zhang X Goncalves R Mosser DM . The isolation and characterization of murine macrophages. Curr Protoc Immunol. (2008) Chapter 14:14.1.1–14.1.14. doi: 10.1002/0471142735.im1401s83, PMID: 19016445 PMC2834554

[28] Lee Y Brenner M Aziz M Wang P . Molecular and subcellular mechanisms of vital macrophage extracellular trap formation. Front Immunol. (2025) 16:1608428. doi: 10.3389/fimmu.2025.1608428, PMID: 40821824 PMC12350110

[29] Taylor PR Martinez-Pomares L Stacey M Lin HH Brown GD Gordon S . Macrophage receptors and immune recognition. Annu Rev Immunol. (2005) 23:901–44. doi: 10.1146/annurev.immunol.23.021704.115816, PMID: 15771589

[30] Raulet DH Gasser S Gowen BG Deng W Jung H . Regulation of ligands for the NKG2D activating receptor. Annu Rev Immunol. (2013) 31:413–41. doi: 10.1146/annurev-immunol-032712-095951, PMID: 23298206 PMC4244079

[31] Denning NL Aziz M Murao A Gurien SD Ochani M Prince JM . Extracellular CIRP as an endogenous TREM-1 ligand to fuel inflammation in sepsis. JCI Insight. (2020) 5:e134172. doi: 10.1172/jci.insight.134172, PMID: 32027618 PMC7141396

[32] Zhou M Aziz M Denning NL Yen HT Ma G Wang P . Extracellular CIRP induces macrophage endotoxin tolerance through IL-6R-mediated STAT3 activation. JCI Insight. (2020) 5:e133715. doi: 10.1172/jci.insight.133715, PMID: 32027619 PMC7141386

[33] Eissmann P Evans JH Mehrabi M Rose EL Nedvetzki S Davis DM . Multiple mechanisms downstream of TLR-4 stimulation allow expression of NKG2D ligands to facilitate macrophage/NK cell crosstalk. J Immunol. (2010) 184:6901–9. doi: 10.4049/jimmunol.0903985, PMID: 20488792

[34] Hamerman JA Ogasawara K Lanier LL . Cutting edge: Toll-like receptor signaling in macrophages induces ligands for the NKG2D receptor. J Immunol. (2004) 172:2001–5. doi: 10.4049/jimmunol.172.4.2001, PMID: 14764662

[35] Jang HR Rabb H . Immune cells in experimental acute kidney injury. Nat Rev Nephrol. (2015) 11:88–101. doi: 10.1038/nrneph.2014.180, PMID: 25331787

[36] Aziz M Brenner M Wang P . Extracellular CIRP (eCIRP) and inflammation. J Leukoc Biol. (2019) 106:133–46. doi: 10.1002/JLB.3MIR1118-443R, PMID: 30645013 PMC6597266

[37] Shimizu J Murao A Lee Y Aziz M Wang P . Extracellular CIRP promotes Kupffer cell inflammatory polarization in sepsis. Front Immunol. (2024) 15:1411930. doi: 10.3389/fimmu.2024.1411930, PMID: 38881891 PMC11177612

[38] Gurien SD Aziz M Jin H Wang H He M Al-Abed Y . Extracellular microRNA 130b-3p inhibits eCIRP-induced inflammation. EMBO Rep. (2020) 21:e48075. doi: 10.15252/embr.201948075, PMID: 31724825 PMC10563445

[39] Antonangeli F Soriani A Cerboni C Sciumè G Santoni A . How mucosal epithelia deal with stress: role of NKG2D/NKG2D ligands during inflammation. Front Immunol. (2017) 8:1583. doi: 10.3389/fimmu.2017.01583, PMID: 29209320 PMC5701928

[40] Xia M Guerra N Sukhova GK Yang K Miller CK Shi GP . Immune activation resulting from NKG2D/ligand interaction promotes atherosclerosis. Circulation. (2011) 124:2933–43. doi: 10.1161/CIRCULATIONAHA.111.034850, PMID: 22104546 PMC3289255

[41] Turner JE Rickassel C Healy H Kassianos AJ . Natural killer cells in kidney health and disease. Front Immunol. (2019) 10:587. doi: 10.3389/fimmu.2019.00587, PMID: 30972076 PMC6443628

