# Supplementary material for: Extracellular CIRP augments inflammation in acute kidney injury via NKG2D-positive macrophages
Source: Front Immunol. 2026 Jan 8;16:1703126. doi: 10.3389/fimmu.2025.1703126 (PMC12823515; doi:10.3389/fimmu.2025.1703126)
Supplement: Supplementary file 1 [file DataSheet1.pdf]

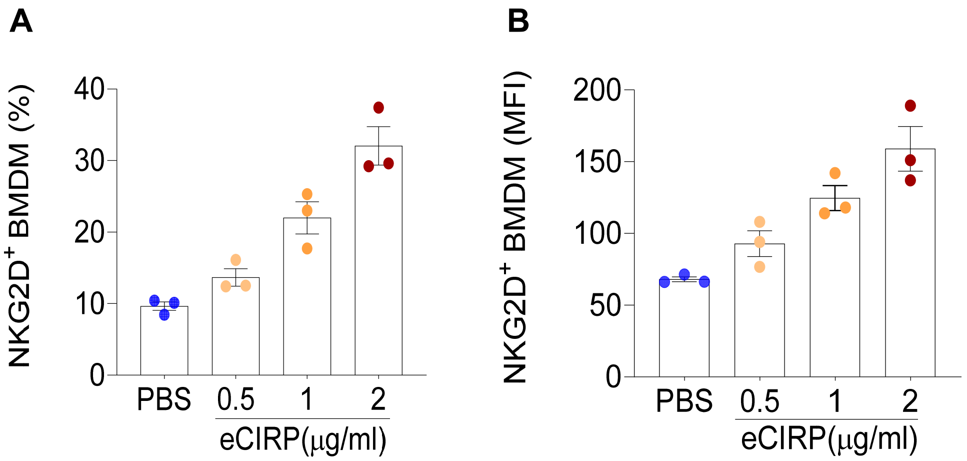

Supplementary Figure 1. eCIRP upregulates the surface expression of NKG2D in cultured primary mouse bone marrow-derived macrophages. Bone marrow-derived macrophages (BMDM) from naïve adult WT mice were isolated and cultured overnight. The cells were then stimulated with 0, 0.5, 1, or 2 μg/ml of recombinant mouse CIRP (eCIRP), followed by detection of NKG2D by flow cytometry. Stimulation with eCIRP dose-dependently increased (**A**) the percentage of NKG2D$^+$ BMDM and (**B**) the MFI of NKG2D in BMDM. *Flow cytometry; n=3/group; mean ± SEM.*
