# Supplementary material for: Extracellular CIRP augments inflammation in acute kidney injury via NKG2D-positive macrophages
Source: Front Immunol. 2026 Jan 8;16:1703126. doi: 10.3389/fimmu.2025.1703126 (PMC12823515; doi:10.3389/fimmu.2025.1703126)
Supplement: Supplementary file 2 [file DataSheet2.pdf]

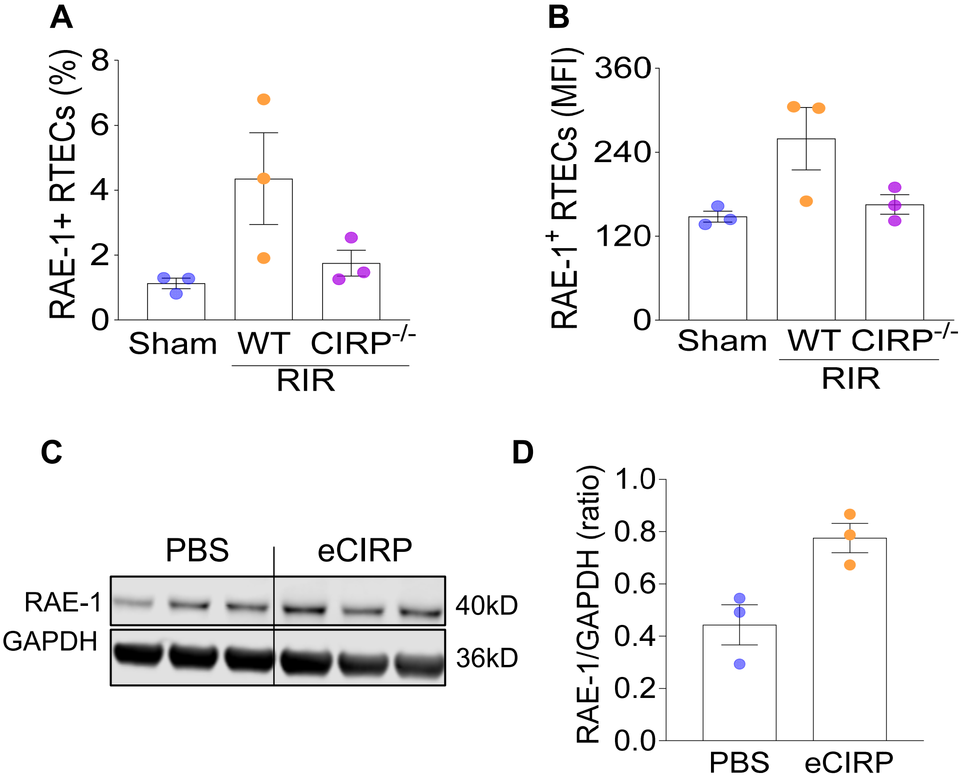

Supplementary Figure 2. eCIRP upregulates RTEC expression of RAE-1 after RIR. RTECs isolated from the kidneys of Sham, RIR WR, and RIR CIRP$^{-/-}$ mice were stained with RAE-1 antibodies. (**A, B**) While RIR increased the percentage of RAE-1^{+} RTECs and the MFI of NKG2D in RTECs from WT mice, no increase was observed in RTECs from CIRP$^{-/-}$ mice. *Flow cytometry; n=3/group; mean ± SEM.* RTECs from naïve adult WT mice were isolated, cultured overnight, and stimulated with eCIRP (1 µg/ml). (**C, D**) 16 hours later, eCIRP increased the protein expression of RAE-1 in RTECs stimulated with eCIRP. *Western blotting, ImageJ quantification; GAPDH, internal control, n=3/group; mean ± SEM.*
