# Supplementary material for: Extracellular CIRP augments inflammation in acute kidney injury via NKG2D-positive macrophages
Source: Front Immunol. 2026 Jan 8;16:1703126. doi: 10.3389/fimmu.2025.1703126 (PMC12823515; doi:10.3389/fimmu.2025.1703126)
Supplement: Supplementary file 3 [file DataSheet3.pdf]

frontiers

The International Society for the Advancement of Cytometry (ISAC) has highlighted the importance of including comprehensive methodological information to ensure data reproducibility and reliability. In line with this, Frontiers in Immunology now requires authors to submit a checklist for manuscripts that involve flow or mass cytometry. This checklist helps standardize the reporting process, improving the quality and transparency of published data. By doing so, we support scientific progress, making it easier for other researchers to replicate and validate experiments.

This form should be submitted with any manuscripts using flow or mass cytometry.

**Sample/specimen/material description**

- [ ] Total blood
- [ ] PBMCs
- [x] Organ digests

Other mouse kidney cells, peritoneal macrophages, bone marrow derived macrophages

Did the samples suffer any treatment before or after incubation with the antibodies?

- [x] Drug stimulation with eCIRP
- [ ] Cell permeabilization ______________________
- [ ] Dye ______________________________________
- [ ] Propidium iodine
- [ ] Not applicable

Other ______________________________________

**Instrument and antibodies**

Name of the Cytometer BD LSR Fortessa flow cytometer

| Antibodies and targets | Fluorochrome/ Metal | Catalog number/Company |
|---|---|---|
| e.g. anti-CD4 | FITC | Cat. XXX/ XXX ltd. |
| Anti-mouse NKG2D | PE | BioLegend Cat.130207 |

| | | |
|---|---|---|
| Anti-mouse F4/80 | APC | BioLegend Cat.123116 |
| | | |
| | | |
| | | |
| | | |
| | | |
| | | |
| | | |
| | | |
| | | |
| | | |
| | | |
| | | |
| | | |
| | | |
| | | |
| | | |
| | | |
| | | |
| | | |
| | | |
| | | |
| | | |
| | | |
| | | |
| | | |
| | | |
| | | |
| | | |

| | | |
|---|---|---|
| | | |
| | | |
| | | |
| | | |
| | | |
| | | |
| | | |
| | | |
| | | |
| | | |
| | | |
| | | |
| | | |
| | | |
| | | |
| | | |
| | | |
| | | |
| | | |
| | | |

**Data analyses**

1. Name of the software FlowJo software (Tree Star, Ashland, OR).
2. Reference gating strategy in the manuscript or supplementary material

Gating strategy in (eg Figure X) Figures 1E, 3C
